# Fine root responses to temporal nutrient heterogeneity and competition in seedlings of two tree species with different rooting strategies

**DOI:** 10.1002/ece3.3794

**Published:** 2018-02-22

**Authors:** Peng Wang, Meng Shu, Pu Mou, Jacob Weiner

**Affiliations:** ^1^ College of Resources and Environmental Sciences Nanjing Agricultural University Nanjing China; ^2^ College of Life Sciences Beijing Normal University Beijing China; ^3^ Department of Plant and Environmental Sciences University of Copenhagen Frederiksberg Denmark

**Keywords:** *Liquidambar styraciflua*, nutrient heterogeneity, *Pinus taeda*, plant–plant interactions, root competition, root foraging, root plasticity, vertical root distribution

## Abstract

There is little direct evidence for effects of soil heterogeneity and root plasticity on the competitive interactions among plants. In this study, we experimentally examined the impacts of temporal nutrient heterogeneity on root growth and interactions between two plant species with very different rooting strategies: *Liquidambar styraciflua* (sweet gum), which shows high root plasticity in response to soil nutrient heterogeneity, and *Pinus taeda* (loblolly pine), a species with less plastic roots. Seedlings of the two species were grown in sandboxes in inter‐ and intraspecific combinations. Nutrients were applied in a patch either in a stable (slow‐release) or in a variable (pulse) manner. Plant aboveground biomass, fine root mass, root allocation between nutrient patch and outside the patch, and root vertical distribution were measured. *L. styraciflua* grew more aboveground (40% and 27% in stable and variable nutrient treatment, respectively) and fine roots (41% and 8% in stable and variable nutrient treatment, respectively) when competing with *P. taeda* than when competing with a conspecific individual, but the growth of *P. taeda* was not changed by competition from *L. styraciflua*. Temporal variation in patch nutrient level had little effect on the species’ competitive interactions. The more flexible *L. styraciflua* changed its vertical distribution of fine roots in response to competition from *P. taeda*, growing more roots in deeper soil layers compared to its roots in conspecific competition, leading to niche differentiation between the species, while the fine root distribution of *P. taeda* remained unchanged across all treatments. *Synthesis*. *L. styraciflua* showed greater flexibility in root growth by changing its root vertical distribution and occupying space of not occupied by *P. taeda*. This flexibility gave *L. styraciflua* an advantage in interspecific competition.

## 1 INTRODUCTION

Heterogeneous distribution of soil resources (i.e., nutrient elements and water) at different spatiotemporal scales is ubiquitous in nature (Farley & Fitter, 1999; Fitter, 1994; Jackson & Caldwell, 1993; Ryel, Caldwell, & Manwaring, 1996). Plants often respond to soil resource heterogeneity through plasticity in root growth. This plasticity consists of morphological changes in root architecture and selective placement of new roots, physiological changes in resource‐uptake rates, and changes in root demography and mycorrhizal associations (Hodge, 2004, 2006; Robinson, 1994). Plasticity in root growth enables plants to optimize their uptake of resources from the soil and thus enhance their performance and fitness (Cahill et al., 2010; Caldwell, Dudley, & Lilieholm, 1992; Chen, Koide, Eissenstat, & van der Heijden, 2018; Fort, Cruz, & Jouany, 2014; Hutchings & de Kroon, 1994).

Plant species vary in their degrees and types of root plasticity. Studies on the effects of soil heterogeneity on root morphology have shown that some species exhibit much stronger selective root placement than other species. The former are called “precise foragers” and the latter, which grow roots less selectively within their rooting zones, are called “scalers,” and they represent two alternative strategies for root foraging (Campbell, Grime, & Mackey, 1991). The contrast between scale and precision of root foraging is often used to characterize rooting strategy of a plant species (Grime, 2007; Einsmann, Jones, Pu, & Mitchell, 1999; but see Kembel, de Kroon, Cahill, & Mommer, 2008). Studies have shown that dominant species usually employ a low‐precision but high‐scale foraging strategy to maximize root foraging area, whereas subordinate species show greater precision (Mommer et al., 2011; Rajaniemi, 2007; Ravenek et al., 2016).

The growth of plants with different types of root plasticity and competitive abilities are affected by different patterns of soil heterogeneity (Hutchings, John, & Wijesinghe, 2003). There is evidence that more precise species are stronger competitors in heterogeneous soils (Bliss, Jones, Mitchell, & Mou, 2002), and species with higher physiological plasticity (i.e., more flexible resource‐uptake rates but less morphological changes) have an advantage under temporally heterogeneous soil conditions (Fransen, de Kroon, & Berendse, 2001). In addition, the presence and identities of competitors can also affect the rooting behavior of plants (Belter & Cahill, 2015; Cahill et al., 2010; Callaway, Pennings, & Richards, 2003; Jackson & Caldwell, 1996; McNickle, Deyholos, & Cahill, 2016), and root responses to soil heterogeneity and to competition from neighbours have been foci of recent studies. The root systems of some plant species avoid overlap, presumably to avoid competition (Brisson & Reynolds, 1994; Einsmann et al., 1999; Mou, Jones, Mitchell, & Zutter, 1995), while other species increase local root proliferation in the presence of neighbours, presumably to gain a competitive advantage (Gersani, Brown, O'Brien, Maina, & Abramsky, 2001). Therefore, it is important to consider both soil heterogeneity and neighboring plants if we want to predict the outcome of root competition among plants.

The aim of this study was to examine the effects of the plastic responses of roots on plant competition when nutrients are distributed heterogeneously in space and time. We used two tree species, an angiosperm *Liquidambar styraciflua* L. (sweet gum) and a gymnosperm *Pinus taeda* L. (loblolly pine). These are two major tree species in the forests of southeastern USA, cooccurring in many habitats, including wet, poorly drained sites (Pataki, Oren, Katul, & Sigmon, 1998). As an early successional species with higher growth rates than *L. styraciflua*,* P. taeda* is known to be tolerant of low soil fertility and highly responsive to changes in resource availability (Griffin, Winner, & Strain, 1995; Samuelson, Stokes, Cooksey, & McLemore, 2001; Williams & Gresham, 2006). It was reported that the *P. taeda* established better than *L. styraciflua* in drier sites (Tolley & Strain, 1984), while other researchers reported that seedlings of both *L. styraciflua* and *P. taeda* had similar responses in root biomass under N‐fertilization (Ludovici & Morris, 1996).

The two species, which belong to different phyla, could have evolved to respond differently to environmental heterogeneity. Some studies showed that they have contrasting root foraging strategies (Einsmann et al., 1999; Mou et al., 1995). When grown individually, *L. styraciflua* shows much stronger morphological plasticity in response to spatial nutrient heterogeneity and greater physiological plasticity to temporal nutrient heterogeneity than does *P. taeda* (Mou, Jones, Tan, Bao, & Chen, 2013; Wang, Mou, & Jones, 2006). However, whether their different root foraging strategies affect competition when the species are growing together remains unclear.

In a common‐garden experiment, we used these two species with contrasting root foraging strategies to investigate the root responses to stable and variable nutrient patches under intra‐ and interspecific competition. We hypothesize that:

1. When competing for patchy nutrients, *L. styraciflua* is competitively superior to *P. taeda* due to its more plastic root morphological responses to spatial nutrient heterogeneity;
2. Compared to stable nutrient patches, temporally variable nutrient patches will reduce root foraging precision of both species but favor competitive advantage of *L. styraciflua*;
3. The two species will adjust their vertical distributions of fine roots under interspecific competition to reduce niche overlap.

## 2 MATERIALS AND METHODS

### 2.1 Experiment setup

**Figure 1:**
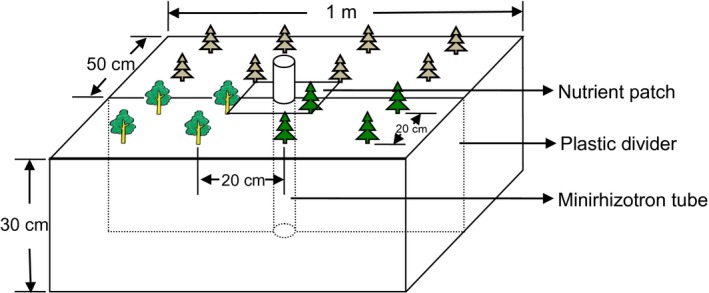
Schematic drawing of the experimental plots. The foreground shows an interspecific competition plot, the background an intraspecific competition plot. The minirhizotron tube was placed in the center, surrounded by the two fertilized patches. The plants at the corners of the fertilized patches were the focal plants

The experiment was carried out in an enclosed experimental field garden (39°57′46″ N, 116°21′25″ E) on the campus of Beijing Normal University, Beijing, China. Eight 1 m^2^ (LWD = 1 × 1 × 0.3 m) experimental sandboxes were constructed. Each box was lined with plastic sheeting to isolate it from the surrounding soil. Before the boxes were filled with construction grade sand, about three hundred holes were punched in the plastic sheets lining the bottom to facilitate drainage. Each sandbox was further divided into two 0.5 m^2^ rectangular plots with a plastic plate, and the sixteen rectangular plots were used as independent experimental units. The sand was washed with HCl‐solution (pH ~ 3) before the boxes were filled. A minirhizotron tube was established at the center of each sandbox to monitor root growth in the two adjacent plots (Figure 1, Figure S1).

Seeds were sowed in germination pots in April 2010 and remained in the greenhouse until early May when the seedlings were about 5 cm tall. The seedlings were then transplanted to the sandboxes, and 20 ml of 1:1,000 commercial fertilizer solution (3.94% NH_4_‐N, 6.05% NO_3_‐N, 10.01% urea N, 20% P_2_O_5_, 20% K_2_O, Peters Professional, the Scotts Co., Marysville, Ohio) was applied to each seedling to promote establishment. There were in total eight plants in each 0.5 m^2^ plot, with two focal plants at the two corners of the central treatment rectangle patch for measuring competition effects, while the other six plants were associates (Figure 1). The planting patterns for examining intra‐ and interspecific competition were: pine–pine (P‐P), sweet gum–sweet gum (S‐S), and sweet gum–pine (S‐P; Figure S1). Seedling that died during the first year was replaced with one that had been growing in the greenhouse.

The plants were irrigated as needed to avoid water stress. In the summer, the plots were covered with shading cloth (25% reduction in light intensity) when the light intensity was high. One year later, all plants in the sandboxes were well established. Before the nutrient treatments were applied, basal area and height of each seedling (Table S1) were measured to determine initial sizes (aboveground biomass) of all the plants based on regression equations fit to 18 and 23 seedlings of *L. styraciflua* and *P. taeda,* respectively, in the greenhouse (Figure S2). In early April 2011, a 10 × 20 cm nutrient patch was established on each side of the plastic divider in the middle of each sandbox (Figure 1), and 12 g slow‐release fertilizer (Osmocote slow‐release fertilizer: 6.8% NO_3_‐N, 8.2% NH_4_‐N, 10% P_2_O_5_, 12% K_2_O, plus micronutrients, Scotts Co., Marysville, Ohio) was applied on the surface of the patch to create a stable nutrient patch. The temporal nutrient patches were created by applying 31 ml of 24:1,000 solution made from the slow‐release fertilizer (equivalent to 0.75 g of the slow‐release fertilizer) to the nutrient patch once per week for 16 weeks. For the patches with stable nutrient or control treatments, the same amount of water was added every week when nutrient solution was applied. After 16 weeks, the granules of the slow‐release fertilizer were all empty, indicating that all nutrients had been released. In a previous study with testing pots, soil nutrient levels in variable nutrient treatments were reduced by about 75% after a week (Mou et al., 2013).

Combining the fertilization and competition treatments gave a 2 × 3 factorial design. The plots were randomly assigned to stable or variable nutrient treatment, eight for each treatment, and the competition pairs (P‐P, S‐S, and S‐P) were randomly assigned to the eight plots of one nutrient treatment with two plots for P‐P, two for S‐S, and four for S‐P. This arrangement resulted in four focal replicates and 12 associate replicates for each species × nutrient treatment combination. There were in total 32 focal plants (16 for *L. styraciflua* and 16 for *P. taeda*) for both nutrient treatments and 96 associated individuals (48 for each species). Each focal plant was considered as one experimental unit in the analyses.

### 2.2 Harvest

In late August 2011, above‐ and belowground parts of the plants were harvested. Before the harvest, the height and basal area of each plant were measured. Soil samples inside (nutrient‐rich) and outside (nutrient‐poor) the fertilized patch in the first layer of each plot were taken before the soil was washed from the roots, and the soil samples were stored at −20°C for laboratory analysis to determine the soil nitrogen contents (Table S2). We did not take soil samples for nutrient analysis during the experiment as it would disturb root growth, especially for roots in the nutrient patches that were relatively small. However, in previous experiments, we and others have found that the slow‐release fertilizer and drips of nutrient solutions resulted in nonsignificant differences of nutrient contents in vertical layers of the soil (Einsmann et al., 1999; Mou et al., 2013; Wang et al., 2006). Soil NH_4_‐N and NO_3_‐N were analyzed using a SEAL Auto‐Analyzer 3 with SEAL AACE software (SEAL Analytical GbmH, Norderstedt, Germany) following the standard procedure (Robertson et al., 1999).

Aboveground parts were cut at the soil surface. Sand in each plot was washed away layer by layer (0–7 cm, 7–14 cm, 14–21 cm, 21–30 cm) to expose the roots. Roots of each individual plant in each layer were harvested and then stored at −20°C before separating the fine roots (diameter <2 mm) and coarse roots (diameter >2 mm). For the two focal plants in each plot, fine and coarse roots of each layer were further divided into in‐and outpatch portions (no roots of associate plants were found in the fertilized patches). All plant samples (leaves, shoots, coarse and fine roots, and root stalks) were oven dried at 65°C to constant weights and weighed.

### 2.3 Data analysis

Because focal plants grown in the same plot were not independent of each other, we used a linear mixed model for our analyses, with species, nutrient treatment, and competition type as fixed factors and sandbox and plot as random factor in a nested structure (plot within sandbox). Before performing the analyses, we examined if there was any influence of initial plant size on the final measurements and found no significant effects (*p *>* *.1).

To test hypotheses 1 and 2, we compared the aboveground biomass, in‐patch fine root mass, total fine root biomass, and root/shoot (R/S) ratio of the focal plants of the two species in different treatments with linear mixed models.

The relative fine root mass difference (RFRMD), which is a measure of the ability of plants to selectively grow their roots in nutrient‐rich patches, was calculated for each focal plant using an equation modified from Mou, Mitchell, and Jones (1997) as the indicator of relative abundance of fine root in nutrient‐rich patch:

RFRMA=(FRI−FRO/3)/TFR,

where FRI is fine root mass in the patch, FRO is the fine root mass outside the patch, and TFR is the total fine root mass. Then, the same model as above was used to compare RFRMD of the two species in different treatments.

To test hypothesis 3, the fine root masses of the two species in different soil layers were compared to examine if they differed in vertical distribution under different treatments. Analyses were performed with R (version 3.3.3) in RStudio (version 1.0.136.0). Linear mixed models were run using package lme4 version 1.1‐7 (Bates, Mächler, Bolker, & Walker, 2015); *p* values were calculated using package lmerTest version 2.0‐20 (Kuznetsova, Brockhoff, & Christensen, 2013).

## 3 RESULTS

**Figure 2:**
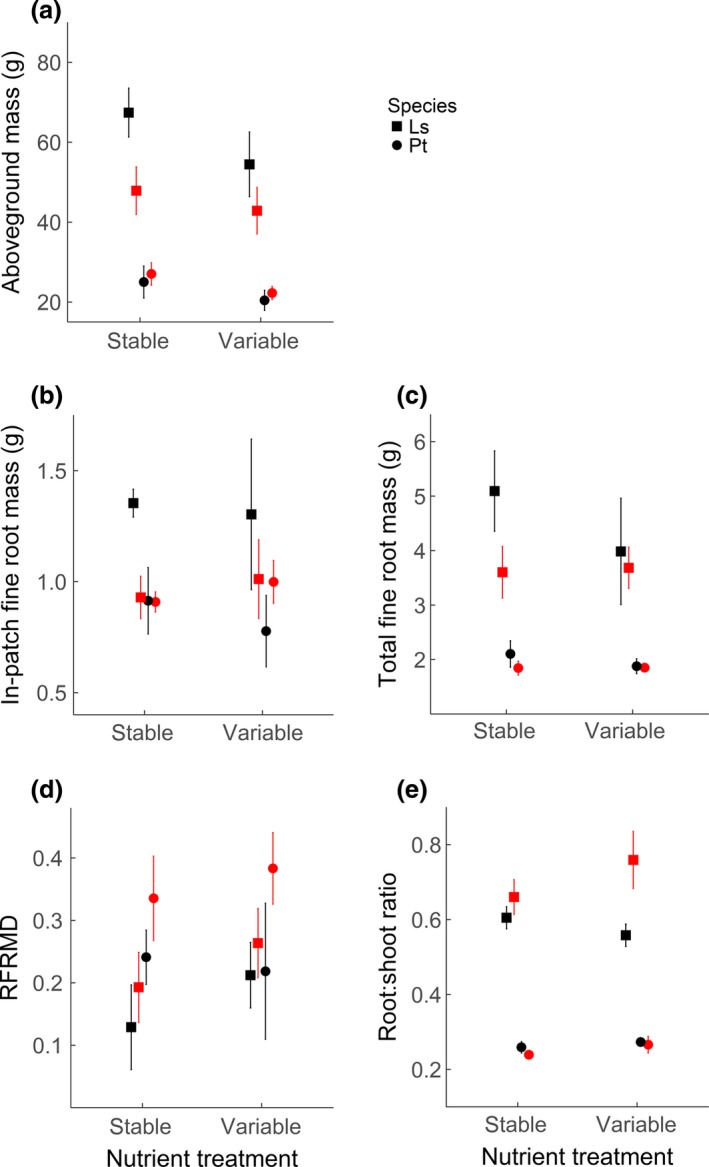
Aboveground biomass (a), in‐patch fine root mass (b), total fine root mass (c), and relative fine root mass difference (RFRMD) (d) of sweet gum and loblolly pine by competition type (inter‐ and intraspecific competition), and the patterns of nutrient supply (stable and variable). Ls, *Liquidambar styraciflua*. Pt, *Pinus taeda*. Symbols in black denote interspecific competition; red denotes intraspecific competition. Error bars represent ±*SE* (*n* = 6)

The aboveground biomass of *L. styraciflua* was significantly higher than that of *P. taeda* in all situations (Table 1). In both stable and variable nutrient treatments, *L. styraciflua* had significantly higher aboveground biomass when competing with *P. taeda* than with conspecifics (*p *=* *.03), while the competition and nutrient treatments did not have significant effects on the biomass of *P. taeda* (Figure 2a). Although not statistically significant, the variable nutrient treatment resulted in a lower average biomass than the stable treatment for both species, especially for *L. styraciflua* (Figure 2a).

**Table 1:** Analyses of effects of species (S), competition (C, inter‐ or intraspecific), treatment (T, stable or variable nutrient), and their combinations on the aboveground biomass, fine root mass in the fertilized patches, total fine root mass, RFRMD, and R/S ratio. Numbers are *F* values in ANOVAs. Significant effects (*p *<* *.1) are marked in bold

Source	*df*	Aboveground biomass	In‐patch fine root biomass	Total fine root biomass	RFRMD	R/S ratio^a^
Species (S)	1	**62.3** ***	**4.5** **	**36.5** ***	**4.6** **	**387.9** ***
Competition (C)	1	2.3	1.1	1.6	2.8	2.2
Treatment (T)	1	2.3	<0.1	0.6	0.9	1.2
S × C	1	**5.5** **	**4.0** *	1.1	0.7	**7.1** **
S × T	1	0.3	<0.1	0.3	0.5	0.3
C × T	1	0.2	0.6	0.7	<0.1	1.9
S × C × T	1	0.3	<0.1	0.4	0.2	0.9

^a^Data were ln transformed to obtain homoscedasticity of residuals.

*p <* *.1; **p <* *.05; ***p <* *.01.

Fine root mass in the nutrient patches showed a significant species effect and a marginally significant species × competition interaction (Table 1). This was due to a higher in‐patch fine root mass of *L. styraciflua* than *P. taeda* in the interspecific competition in both nutrient treatments (*p *=* *.06). For intraspecific competition, the in‐patch fine root masses of the two species were similar (Table 1, Figure 2b). In general, total fine root mass was significantly higher for *L. styraciflua* than *P. taeda*, but total fine root mass of both species did not change significantly among the treatments (Table 1, Figure 2c).

Relative fine root mass difference was significantly lower for *L. styraciflua* than *P. taeda* (Table 1, Figure 2d), and RFRMD did not change significantly in different treatments. R/S ratios of *L. styraciflua* were significantly higher than that of *P. Taeda* across all treatments (Table 1, Figure 2e). In addition, the R/S ratios of *L. styraciflua* were significantly lower when competing with *P. taeda* than competing with conspecifics in variable nutrient treatment (*p *=* *.02, Figure 2e). The R/S ratios of *P. taeda* were similar across all nutrient and competition treatments (Table 1, Figure 2e).

**Figure 3:**
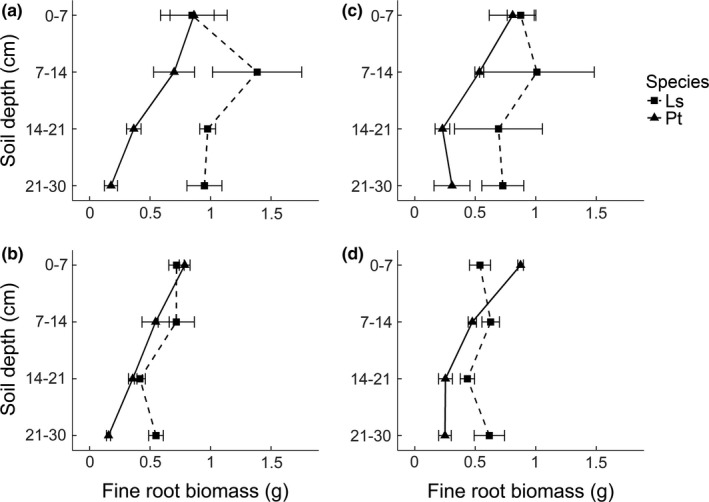
Distribution of fine root mass of focal plants by soil layers under the stable (a, b) and variable (c, d) nutrients, interspecific (a, c) and intraspecific (b, d) competition conditions. Ls, *Liquidambar styraciflua*. Pt, *Pinus taeda*. Error bars represent ±*SE* (*n* = 6)

The vertical fine root distribution of *P. taeda* was relatively constant across all treatments, while that of *L. styraciflua* differed considerably between inter‐ and intraspecific competition (Figure 3). In the topsoil layer, *L. styraciflua* and *P. taeda* had similar amounts of fine roots when they were competing with each other, while under intraspecific competition, *L. styraciflua* grew fewer roots than *P. taeda* did (9% and 38% fewer in stable and variable nutrient patches, respectively; Table 2, Figure 3). In general, *L. styraciflua* proliferated more fine roots than *P. taeda* in the deeper layers (Figure 3). When competing with *P. taeda*,* L. styraciflua* grew more roots than *P. taeda* in the third layers (160% and 200% more in stable and variable nutrient patches, respectively; Table 2, Figure 3), although the increase was only marginally significant. A similar trend was also found in the second layer (Figure 3). The variable nutrient treatment significantly reduced fine root mass of both species in the third layer compared to stable nutrient treatment (Table 2). In general, vertical root distribution patterns inside and outside the nutrient patches were similar both species across all treatments (Figure S3).

**Table 2:** Analyses of effects of species (S), competition (C, inter‐ or intraspecific), and treatment (T, stable or variable nutrient) on the fine root mass in different soil layers. Data were ln transformed to obtain normal distribution and homoscedasticity of residuals. Numbers are *F* values in ANOVAs. Significant effects (*p *<* *.1) are marked in bold

Source	*df*	0–7 cm	7–14 cm	14–21 cm	>21 cm
Species (S)	1	1.7	**7.7** **	**16.5** ***	**47.4** ***
Competition (C)	1	<0.1	1.5	1.6	0.4
Treatment (T)	1	0.1	1.1	**6.0** **	0.6
S × C	1	**6.5** **	0.8	**3.2** *	0.7
S × T	1	1.0	0.3	0.2	1.4
C × T	1	<0.1	0.5	1.9	0.6
S × C × T	1	2.7	0.3	0.8	0.3

*p <* *.1; **p <* *.05; ***p <* *.01.

## 4 DISCUSSION

Our results indicate that *L. styraciflua* performed better in competition with *P. taeda*, with the aboveground biomass of *L. styraciflua* increased by 42% and 27% in interspecific competition in the stable and variable nutrient treatments, respectively, compared to that in intraspecific competition (Figure 2a). This is consistent with our first hypothesis. However, *P. taeda* did not show growth reduction when competing with *L. styraciflua* compared to *P. taeda* in conspecific competition. The lack of response of *P. taeda* to the competition from *L. styraciflua* may be due to the complementarity of their root distribution patterns when growing together.

### 4.1 Competitive ability of the two species

Many studies have shown that the ability to occupying nutrient‐rich patches quickly can play a more important role than other root traits in plant root competition (Fort et al., 2014; McNickle et al., 2016; Mommer, van Ruijven, Jansen, van de Steeg, & de Kroon, 2012; Ravenek et al., 2016; Semchenko, Lepik, Abakumova, & Zobel, 2017). Our results showed that *L. styraciflua* had an advantage in terms of aboveground biomass when competing with *P. taeda*, possibly through the former's higher root mass, which could give the species more access to nutrients in the soil. The in‐patch fine root masses of both species were similar under intraspecific competition, while *L. styraciflua*, which showed high root plasticity in previous studies (Mou et al., 2013; Wang et al., 2006), increased its fine root mass by 30–45% in the interspecific competition compared to intraspecific competition, but the in‐patch fine root mass of *P. taeda* remained unchanged. The total fine root mass showed a similar pattern. It is also possible that *L. styraciflua* grew bigger root system under interspecific than conspecific competition because the latter is more severe. Although the two species had similar fine root mass in the nutrient patches in conspecific competition, roots of *L. styraciflua* are thinner than that of *P. taeda* (Mou et al., 2013; Wang et al., 2006), implying that, with similar root mass, root length density of the former is higher than the latter, which is supported by our minirhizotron observations (Figure S4). Root length density may be a better indicator of competitiveness than root mass (Mommer et al. (2011). If this is true, thinner roots of *L. styraciflua* mean that, for a given root mass, intensity of root competition is higher for *L. styraciflua* than for *P. taeda*. Furthermore, the role of mycorrhizal fungi in the uptake efficiency per root mass/length needs further studies, as mycorrhizal association for nutrient uptake is stronger in *P. taeda* than in *L. styraciflua* (Constable, Bassirirad, Lussenhop, & Zerihun, 2001).

Although *P. taeda* seemed to be inferior in terms of root growth, its growth was not reduced by *L. styraciflua*, either aboveground or belowground. This could be due to the short duration of this experiment (16 weeks of the nutrient treatments) relative to the life spans of the species, making the effects of competition for nutrients too small to appear. In addition, species with lower‐specific root length and less branched roots may be better at tolerating competition (Semchenko et al., 2017), as these traits can be associated with high levels of mycorrhizal colonization (Koziol & Bever, 2015; Maherali, 2014). Previous studies have shown that roots of *P. taeda* are lower in specific root length and less branched than those of *L. styraciflua* (Einsmann et al., 1999; Mou et al., 2013; Wang et al., 2006), and thus, *P. taeda* may be able to handle the competition from its neighbors if the competition intensity is low, such as at the early stage of seedling establishment in this study.

### 4.2 Scale and precision of root foraging

In our study, *P. taeda* demonstrated higher RFRMD values (i.e., root foraging precision) than *L. styraciflua*, especially under intraspecific competition (Figure 2). This finding differed from previous reports that *L. styraciflua* is a precise forager with higher morphological root plasticity than *P. taeda* (Einsmann et al., 1999; Wang et al., 2006). Campbell et al. (1991) hypothesized that a superior competitor dominates in foraging scale, proliferating roots in both nutrient‐rich and nutrient‐poor patches, while the inferior competitor focuses on foraging precision by allocating much of the roots in nutrient patches. Our results seem to be consistent with this hypothesis, as the competitively superior species, *L. styraciflua*, dominated both in and out of the patches, although it has been categorized as a precise forager; while *P. taeda* confined most of its roots in the patches, although it is thought to be a foraging scaler.

There are two possible explanations for these changes in the precision of root foraging. The first is that interspecific competition may change root growth pattern of plants. Some plant species tend to grow roots aggressively toward an interspecific competitor to gain competitive advantage (Bartelheimer and Beyschlag (2006). *Plantago lanceolate* overproduced roots in both nutrient‐rich and nutrient‐poor soils when growing with *Festuca rubra*, making *P. lanceolate* the more superior species in interspecific competition (Padilla et al. (2013). Some species appear to prioritize information about neighbors higher than information about nutrients in the placement of roots (McNickle et al. (2016), which means such plants will always alter their root foraging behavior when competing with a neighbor. The second possible explanation is that patch saturation by roots may change the species’ rooting patterns. Minirhizotron data revealed fine root saturation of the patches as the root growth leveled off weeks before harvest (Figure S4). It is likely that fine roots, especially those of *L. styraciflua,* were forced to grow out of the nutrient patches when the nutrient‐rich patches became fully occupied. The two possible explanations are not exclusive, and both could function at the same time to change plant rooting patterns.

Contrary to our second hypothesis, temporal nutrient heterogeneity had little effect on root foraging precision and the outcome of competition in our experiment. Variable nutrient treatment tended to decrease aboveground biomass of both species (Figure 2a). As nutrients in the variable treatment were applied in solution, we cannot rule out the possibility that nutrients were leached more than in the stable nutrient treatment, resulting in fewer accessible nutrients and thus lower biomass growth.

### 4.3 Vertical niche differentiation and implications for long‐term competition between the two species

The vertical distribution of fine roots supported our third hypothesis. Under intraspecific competition, the two species exhibited similar root vertical distribution pattern, but in competition with *P. taeda*,* L. styraciflua* changed its vertical distribution pattern and grew more roots in deeper soil layers (Figure 3), resulting in niche differentiation in vertical root distribution between the two species. Previous studies have also shown vertical niche differentiation when different species were growing together (Brassard et al., 2013; Laclau et al., 2013; McKane et al., 2002; Wang et al., 2017), resulting in root overyielding (Brassard et al., 2013; Laclau et al., 2013). We did not find significant root overyielding in our study, perhaps due to the high variability in our results. Some studies did not find horizontal or vertical root niche differentiation (McNickle et al., 2016; Mommer et al., 2010; Valverde‐Barrantes, Smemo, Feinstein, Kershner, & Blackwood, 2015). It has been hypothesized that complementary effects of diversity on root biomass could depend on phylogenetic relatedness within root neighborhoods (Valverde‐Barrantes et al., 2015). Belter and Cahill (2015) proposed the location‐sensitivity rooting strategy, in which case plants adjust their root horizontally and vertically when encountering neighbors, thus enhancing coexistence. Our data suggest that *L. styraciflua* shows such location‐sensitivity.

The root length increment was larger for *L. styraciflua* as revealed by minirhizotron data (Figure S4), particularly in the deeper layers, suggesting that this species can fill a soil volume more quickly than *P. taeda*. The ability of quickly filling soil volume and the high flexibility in the root growth of *L. styraciflua* may enable it to respond to changes in environmental conditions and neighboring plants and thus take advantage of resources that other plants have not yet reached, giving it a competitive advantage over other plants that respond less. Due to the relative short duration of this experiment, the competitive advantages of *L. styraciflua* over *P. taeda* may have been limited and the growth of *P. taeda* may not yet have been significantly affected by interspecific competition. The increased growth of *L. styraciflua* is very likely to inhibit *P. taeda* in a longer term, however, either through shoot competition or through root competition, and thus outcompete the latter.

## 5 CONCLUSIONS

Our results revealed rooting foraging patterns, including root selective placement and vertical root distribution, in competition between two tree species with contrasting rooting strategies. *L. styraciflua* had an advantage expressed in increased aboveground growth, while *P. taeda* showed little change in growth. The more flexible *L. styraciflua* changed its vertical fine root distribution in response to interspecific competition, showing niche differentiation compared to the more fixed rooting pattern of *P. taeda*. We conclude that *L. styraciflua* is a competitively superior species, in part because of its flexibility in root growth, and would likely outcompete *P. taeda* when grow together over the long term.

## CONFLICT OF INTEREST

None declared.

## AUTHOR CONTRIBUTIONS

PW and PM conceived the experiment; PW collected the data; PW and PM analyzed the data; and PW, MS, PM, and JW wrote the manuscript. All authors contributed substantially to the drafts and gave final approval for publication.
