# Supplementary material for: Fine root responses to temporal nutrient heterogeneity and competition in seedlings of two tree species with different rooting strategies
Source: Ecol Evol. 2018 Feb 22;8(6):3367–75. doi: 10.1002/ece3.3794 (PMC5869361; doi:10.1002/ece3.3794)
Supplement: Supplementary file 1 [file ECE3-8-3367-s001.docx]

**Wang et al. 2018.** Fine root responses to temporal nutrient heterogeneity and competition in seedlings of two tree species with different rooting strategies. *Ecology and Evolution.*

**Supplementary information**

Table S1. Initial and final plant size of focal plants in all plots.

| Species | Treatment | Plot no. | | Initial basal area (mm^2^) | | Initial height (cm) | | Final basal area (mm^2^) | | Final height (cm) | |
| --- | --- | --- | --- | --- | --- | --- | --- | --- | --- | --- | --- |
| Sweetgum | Inter-specific; stable patches | 1 | 3.54 | | 13.4 | | 16.70 | | 88.6 | |  |
|  |  | 5 | 4.46 | | 14.4 | | 19.06 | | 89.6 | |  |
|  |  | 10 | 4.34 | | 16.4 | | 16.78 | | 89.7 | |  |
|  |  | 11 | 4.34 | | 9.3 | | 16.08 | | 73.3 | |  |
|  | Inter-specific; variable patches | 4 | 4.10 | | 16.4 | | 20.47 | | 81.5 | |  |
|  |  | 13 | 2.82 | | 10.9 | | 13.68 | | 74.7 | |  |
|  |  | 15 | 5.38 | | 16.5 | | 17.68 | | 69.5 | |  |
|  |  | 16 | 3.94 | | 16.9 | | 12.10 | | 62.8 | |  |
|  | Intra-specific; stable patches | 19 | 4.56 | | 17.1 | | 15.51 | | 73.5 | |  |
|  |  | 19 | 3.94 | | 16.9 | | 14.97 | | 92.6 | |  |
|  |  | 22 | 3.96 | | 17.2 | | 15.01 | | 70.2 | |  |
|  |  | 22 | 3.82 | | 11.3 | | 14.42 | | 55.4 | |  |
|  | Intra-specific; pulse patches | 18 | 4.88 | | 21.5 | | 14.61 | | 69.5 | |  |
|  |  | 18 | 4.92 | | 20.5 | | 12.35 | | 58.1 | |  |
|  |  | 23 | 5.34 | | 9.3 | | 15.84 | | 58.0 | |  |
|  |  | 23 | 3.12 | | 7.6 | | 15.16 | | 61.5 | |  |
| Loblolly pine | Inter-specific; stable patches | 1 | 3.18 | | 16.5 | | 8.67 | | 55.4 | |  |
|  |  | 5 | 4.00 | | 20.8 | | 9.85 | | 62.4 | |  |
|  |  | 10 | 5.04 | | 17.2 | | 9.90 | | 42.3 | |  |
|  |  | 11 | 3.34 | | 16.5 | | 7.31 | | 51.6 | |  |
|  | Inter-specific; variable patches | 4 | 3.38 | | 18.8 | | 8.71 | | 52.7 | |  |
|  |  | 13 | 2.62 | | 17.6 | | 8.13 | | 50.1 | |  |
|  |  | 15 | 3.70 | | 17.1 | | 11.19 | | 66.8 | |  |
|  |  | 16 | 3.14 | | 15.8 | | 8.28 | | 49.7 | |  |
|  | Intra-specific; stable patches | 20 | 2.66 | | 19.0 | | 10.49 | | 54.3 | |  |
|  |  | 20 | 2.74 | | 17.1 | | 9.37 | | 54.2 | |  |
|  |  | 21 | 2.32 | | 17.4 | | 8.97 | | 57.1 | |  |
|  |  | 21 | 2.88 | | 9.7 | | 9.76 | | 44.8 | |  |
|  | Intra-specific; pulse patches | 17 | 3.40 | | 18.2 | | 8.54 | | 54.4 | |  |
|  |  | 17 | 3.50 | | 17.6 | | 10.42 | | 55.1 | |  |
|  |  | 24 | 3.20 | | 17.5 | | 9.96 | | 58.9 | |  |
|  |  | 24 | 2.76 | | 14.5 | | 9.49 | | 46.3 | |  |

Table S2. Concentrations of ammonium and nitrate nitrogen inside and outside the nutrient patches in stable and variable nutrient treatments. Values are mean ± SE.

|  | Stable nutrient treatment | |  | Variable nutrient treatment | | |
| --- | --- | --- | --- | --- | --- | --- |
|  | In-patch | Out-patch |  | In-patch | Out-patch | |
| NH_4_-N | 42.11±1.05 | 37.31±4.24 |  | 44.55±0.34 | 41.62±1.08 | |
| NO_3_-N | 0.26±0.03 | 0.49±0.17 |  | 0.35±0.03 | 0.30±0.02 | |
| Total N | 42.38±1.04 | 37.80±4.09 |  | 44.90±0.31 | 41.93±1.10 | |
| Unit: mg N g^−1^ dry soil | | | | | |  |


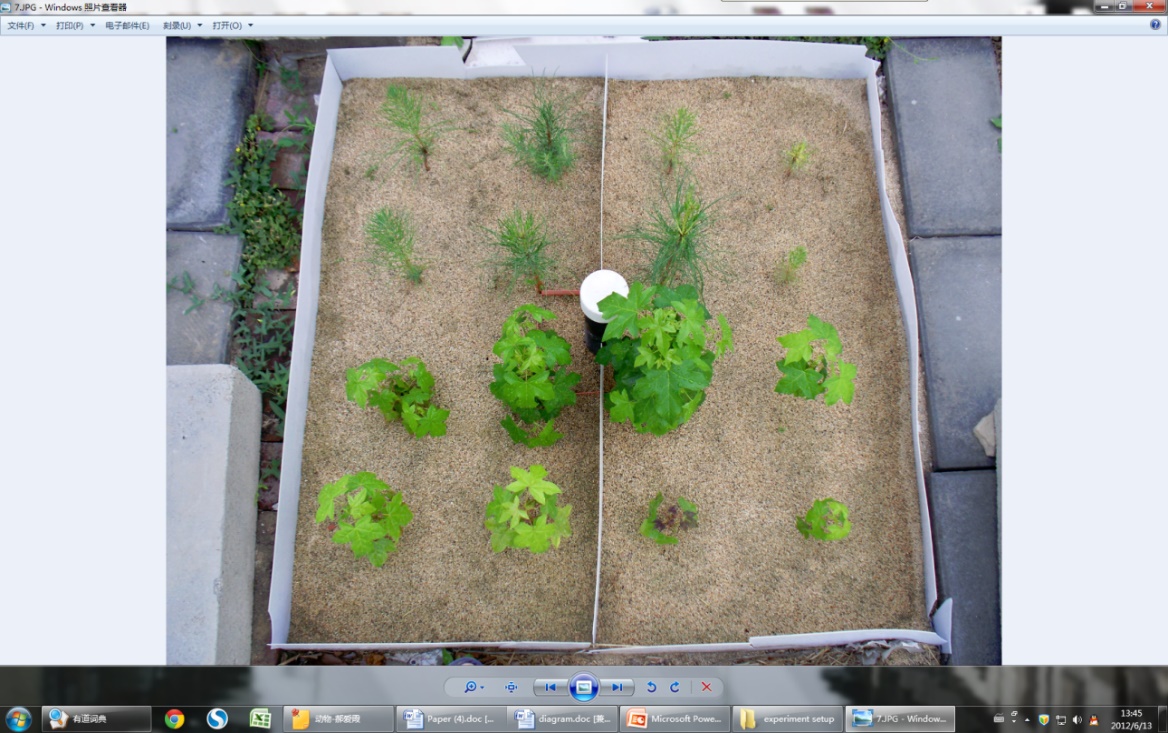

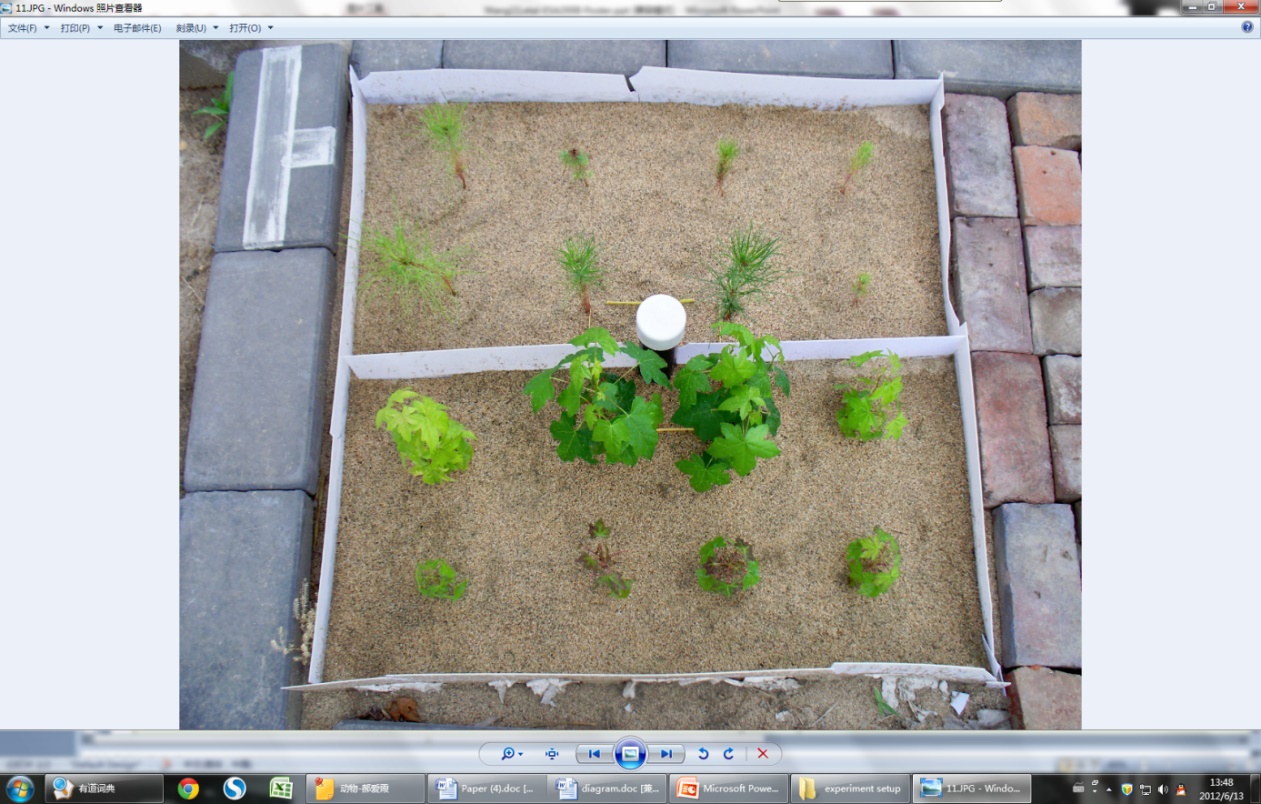


Fig. S1. Photographs of two inter-specific competition plots (left) and two intra-specific competition plots (right). The minirhizotron tube was placed in the centre, surrounded by the two fertilized patches. The plants at the corners of the fertilized patches were the focal plants. Photos were taken one month before the harvest.


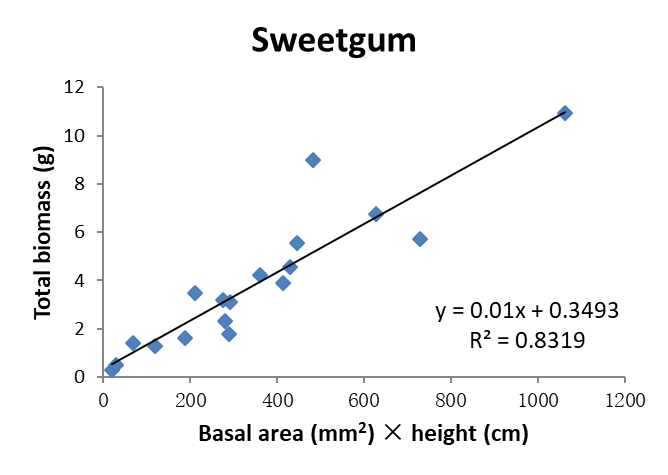

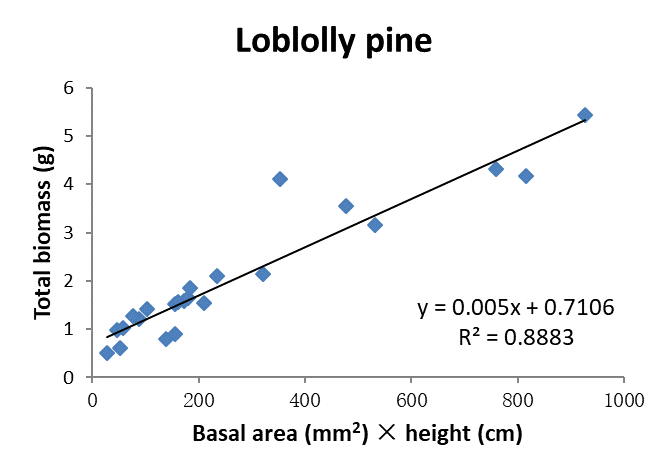


Fig. S2. Regression relationships used to estimate initial biomass of focal plants. They were fit to 18 and 23 seedlings of sweetgum (*Liquidambar styraciflua*) and loblolly pine (*Pinus taeda*), respectively, in the greenhouse.


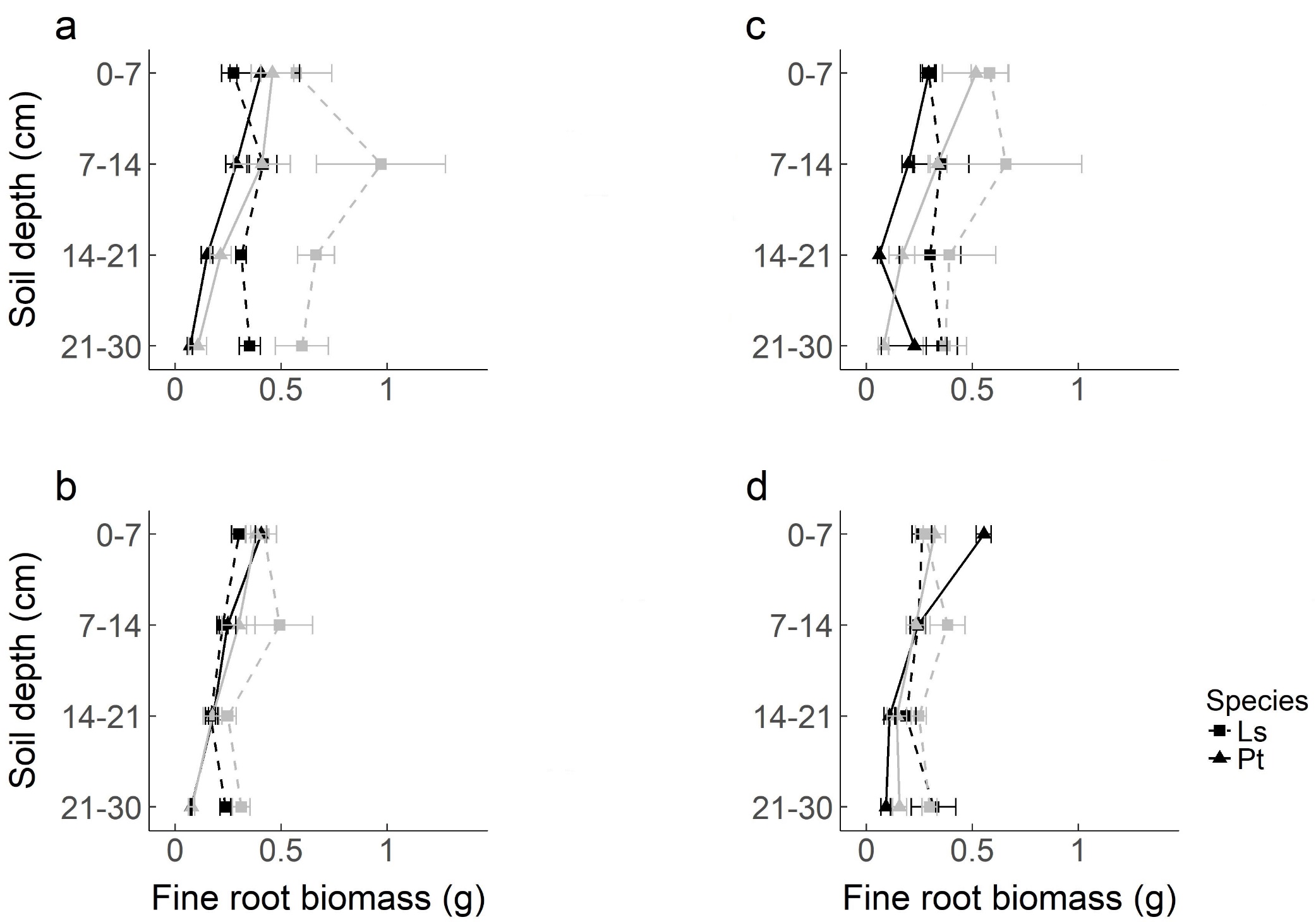


Fig. S3. Fine root mass in and out of the nutrient patches of focal plants by soil layer under the stable (a, b) and variable (c, d) nutrient, inter-specific (a, c) and intra-specific (b, d) competition conditions. Ls, *Liquidambar styraciflua*. Pt, *Pinus taeda*. Shapes in black denote in-patch fine roots; shapes in grey denote out-patch fine roots. Error bars represent ± SE (n = 6).


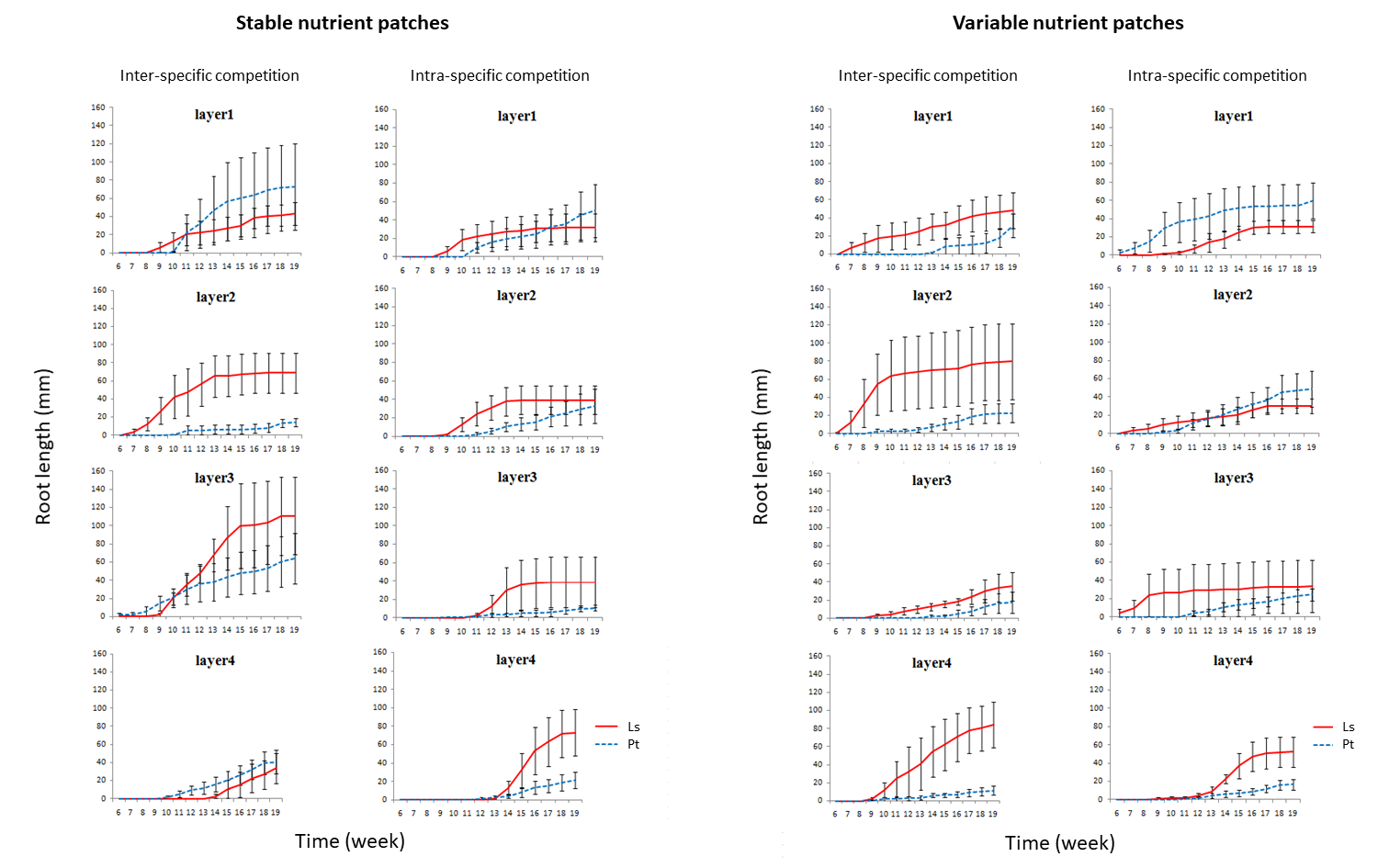
Fig. S4. Root length increment of *Liquidambar styraciflua* and *Pinus taeda* in the four treatments during the experiment.
